# 

**Published:** 1902-12

**Authors:** 


NOW IS THE TIME TO RENEW SUBSCRIPTIONS FOR 1903

December, 1902. Vol. XXIII. No. 12.

### THE INTERNATIONAL DENTAL JOURNAL.

JAMES TRUMAN, D.D.S., Editor,
PHILADELPHIA.

*COLLABORATORS:*

CHARLES P. BRIGGS, A.B., M.D., D.M.D.,
BOSTON.

JOHN A. McCLAIN, D.D.S.,
PHILADELPHIA.

WILLIAM H. POTTER, A.B., D.M.D.,
BOSTON.

LIBRARY
SURGEON GENERAL'S OFFICE
15-DEC-1902

### Summary of Contents.

(*For details, see p. ii.*)

ORIGINAL COMMUNICATIONS.
REVIEWS OF DENTAL LITERATURE.
REPORTS OF SOCIETY MEETINGS.
EDITORIAL.
MISCELLANY.
CURRENT NEWS.
DOMESTIC CORRESPONDENCE.

*$2.50 a Year, in Advance. Single Copies, 25 Cents.*

PUBLISHED MONTHLY BY

The International Dental Publication Company,

227-231 SOUTH SIXTH ST., PHILADELPHIA.

EDITORIAL OFFICE, 4505 CHESTER AVENUE, PHILADELPHIA, PA.

Printed by J. B. Lippincott Company, Philadelphia.

Entered at the Post-Office at Philadelphia, and admitted for transmission through the mails at second-class rates

